# An Unusual Case of Post-Operative Functional Stridor After Emergence From General Anesthesia

**DOI:** 10.7759/cureus.41757

**Published:** 2023-07-12

**Authors:** Prakash Deb, Jabed Ahmed, Hanifa Akhtar, Kaustuv Dutta, Mohd Yunus

**Affiliations:** 1 Anesthesiology and Critical Care, All India Institute of Medical Sciences, Guwahati, Guwahati, IND; 2 Critical Care Medicine, Christian Medical College, Vellore, Vellore, IND; 3 Otorhinolaryngology, All India Institute of Medical Sciences, Guwahati, Guwahati, IND; 4 Anesthesiology and Critical Care, North Eastern Indira Gandhi Regional Institute of Health and Medical Sciences, Shillong, IND; 5 Emergency Medicine and Trauma, All India Institute of Medical Sciences, Bhopal, Bhopal, IND

**Keywords:** functional stridor, laryngeal edema, conversion disorder, stridor, laryngospasm, post-anaesthesia

## Abstract

Post-anesthesia stridor due to laryngospasm, laryngeal edema, or any other organic cause is a life-threatening condition requiring immediate intervention. The very rare functional stridor or psychogenic stridor following emergence from general anesthesia may sometimes mimic stridor due to an organic cause, but it is neither fatal nor require immediate airway management. However, if the condition is not diagnosed timely, it may lead to unnecessary manipulation of the airway, such as endotracheal intubation or tracheostomy. We report herein a case of functional stridor in a 48-year-old woman who underwent abdominal-perineal resection for carcinoma rectum. The case was timely diagnosed by the attending anesthetist, and the patient recovered spontaneously, thus avoiding any unindicated airway handling and its associated complications.

## Introduction

Functional or psychogenic stridor is a conversion disorder where stridor happens after stress or conflict, and there is an absence of any somatic cause, resulting in significant impairment. Functional stridor following emergence from general anesthesia is very rare. Stridor after extubation is a sign of underlying life-threatening upper airway obstruction such as laryngospasm or laryngeal edema, which needs immediate intervention. However, functional stridor may misguide the anesthetist, leading to unwanted invasive manipulation to re-establish the airway, resulting in cardiac and respiratory complications, mortality, prolonged stay, and cost. [1-3] We report herein a case of 48 year-old woman who underwent open abdominal-perineal resection (APR) for carcinoma of the rectum and developed functional stridor post-operatively following extubation. This was timely diagnosed by the anesthetist, and an unanticipated airway intervention was avoided.

## Case presentation

A 48-year-old woman was posted for open APR for carcinoma of the rectum. She had received neo-adjuvant chemotherapy and radiotherapy. Her body mass index was 17.1 kg/m^2^ and had no comorbidity. Pre-anesthesia evaluation revealed that she was very anxious about her surgery and prognosis of the disease for which she was prescribed anxiolytics and counselled multiple times. However, there was no other significant medical, surgical, or drug history. No gross abnormality was noted during airway assessment and physical examination. All pre-operative laboratory investigations and computed tomography scan of the thorax were within the normal range.

General anesthesia was administered along with epidural analgesia. Pre-operatively, an epidural catheter was placed in the T10-11 level using a Tuohy needle of 18G in size using the loss-of-resistance technique, and the catheter was fixed at 10 cm. Induction was performed using injection fentanyl 70 micrograms intravenous, injection propofol 80 mg intravenous, and injection rocuronium 40 mg intravenous. Intubation was performed orotracheally using an endotracheal tube with an internal diameter of 7.5 mm with the help of direct laryngoscope (MAC-3 blade). Intubation was smooth without requiring any manipulation or external assistance. Anesthesia was maintained using oxygen and air with sevoflurane. Analgesia was achieved using continuous infusion of bupivacaine 0.125% and fentanyl 2 microgram/ml via the epidural route. Injection ondansetron 4 mg was administered intravenously for post-operative nausea and vomiting prevention. Paracetamol 1 gm and ketorolac 30 mg was administered slowly intravenously toward the end of the procedure for post-operative analgesia. Blood loss was approximately 450 mL and required one unit of packed red blood cell transfusion. The patient was hemodynamically stable throughout the perioperative period. Reversal was achieved using injection neostigmine 2.5 mg and injection glycopyrrolate 0.4 mg intravenous. Duration of surgery was approximately 4 hours. Extubation was performed once the patient became awake and the return of neuro-muscular power was adequately established. The patient was hemodynamically stable, pain-free, and comfortable.

While shifting the patient from the operation theater to the post-anesthesia care unit, she developed sudden inspiratory stridor and high respiratory rate (30/min). Clinically, there was no desaturation, and the chest was clear with bilateral air entry on auscultation. An anesthesia mask was placed with 100% oxygen supplementation and two positive pressure breaths were delivered, which was followed by cessation of stridor. The patient was fully conscious and oriented, obeying commands, and moving all limbs. It was decided to shift the patient to the intensive care unit where she developed similar episodes twice at 5- to 10-minute interval. The patient was closely monitored, and all measures to manage laryngospasm, re-intubation, and ventilation were ready, but no active interventions were required. The episodes lasted for approximately 10 seconds each time and subsided spontaneously. The ENT service was consulted during this stay. A fiberoptic laryngoscopy under local anesthesia could not reveal any structural abnormality. No further episodes happened for another 24 hours.

The patient was shifted to the ward where proper neurological and psychiatric evaluation was done and a provisional diagnosis of functional stridor was made. The admission course was uneventful, and the patient was discharged after 14 days of hospital stay with an advice to follow up in the psychiatry and neurology outpatient department in the hospital after four weeks.

## Discussion

Stridor is a high-pitched sound due to turbulent airflow through a narrowed airway. Due to tonal variation of stridor, it may mimic wheeze or stertor. Laryngospasm and laryngeal edema are commonly implicated in a case of acute stridor following extubation. Laryngospasm may be triggered by multiple surgical and anesthesia-related factors such as inadequate anesthetic depth during airway manipulation, hypersensitive airway, upper respiratory tract infection, certain types of surgery such as adenoidectomy and tonsillectomy [4]. Laryngospasm following extubation may be life-threatening if not detected and corrected immediately. In our case, the patient was extubated after reversing the neuro-muscular block (confirming that clinically) and with peripheral nerve stimulator. Our patient was totally conscious, oriented, and responsive to commands. Laryngeal edema following extubation will not resolve spontaneously and immediately, as happened in our case. Severe anxiety, chronic pain, or emotional stress may predispose to such events. Acute stridor in this post-operative period without any clinical deterioration and in the absence of any organic pathology, upper airway infection, or pre-existing neuropsychiatric illness could be a case of functional neurological symptom disorder (FNSD). Functional stridor is a diagnosis of exclusion and needs a high clinical suspicion. The treatment is supportive and reassurance.

There are some case reports of post-operative FNSD or conversion disorder with presentations such as tetraplegia, rigor, delirium, torticollis, lower limb paralysis, tongue weakness, and hemiparesis; however, the occurrence of functional stridor following anesthesia is very rare [5-9]. Psychogenic stridor, which is also known by the name of Munchausen stridor, factitious asthma, functional upper airway obstruction, or hysterical stridor is very rarely observed in post-operative cases. One case of post-operative functional stridor was described in a 13-year-old female patient, which progressed to the features of unconsciousness and hemiparesis. No neurological or ENT cause could be evaluated in that case after a proper investigation including MRI brain and nasal fiberoptic scope examination [10]. Indirect laryngoscopy during the acute event of functional stridor may demonstrate a paradoxical vocal cord motion [3]. Arterial blood gases during acute phase may demonstrate the absence of hypoxemia and hypercarbia, though it is not a consistent feature [11].

## Conclusions

Functional stridor is very rare in the post-operative period. The diagnosis needs a high index of suspicion and exclusion of life-threatening organic causes of airway obstruction and narrowing. Failure to identify functional stridor may lead to unnecessary interventions such as laryngoscopy, intubation, and tracheostomy. It is essential to keep FNSD as a differential in cases of post-anesthesia stridor to avoid any invasive manipulation of the airway.
